# A Study of Insulin Resistance by HOMA-IR and its Cut-off Value to Identify Metabolic Syndrome in Urban Indian Adolescents

**DOI:** 10.4274/Jcrpe.1127

**Published:** 2013-12-12

**Authors:** Yashpal Singh, MK Garg, Nikhil Tandon, Raman Kumar Marwaha

**Affiliations:** 1 Command Hospital (Southern Command), Department of Endocrinology, Pune, India; 2 All Indian Institute of Medical Sciences, Department of Endocrinology, New Delhi, India; 3 International Life Sciences, Scientific Advisor (Projects), New Delhi, India

**Keywords:** insulin resistance, metabolic syndrome, HOMA-IR, adolescents

## Abstract

**Objective:** Insulin resistance (IR) and associated metabolic abnormalities are increasingly being reported in the adolescent population. Cut-off value of homeostasis model of assessment IR (HOMA-IR) as an indicator of metabolic syndrome (MS) in adolescents has not been established. This study aimed to investigate IR by HOMA-IR in urban Indian adolescents and to establish cut-off values of HOMA-IR for defining MS.

**Methods:** A total of 691 apparently healthy adolescents (295 with normal body mass index (BMI), 205 overweight, and 199 obese) were included in this cross-sectional study. MS in adolescents was defined by International Diabetes Federation (IDF) and Adult Treatment Panel III (ATP III) criteria. IR was calculated using the HOMA model.

**Results:** Mean height, waist circumference (WC), waist/hip ratio (WHR), waist/height ratio (WHtR), and blood pressure were significantly higher in boys as compared to girls. The HOMA-IR values increased progressively from normal weight to obese adolescents in both sexes. Mean HOMA-IR values increased progressively according to sexual maturity rating in both sexes. HOMA-IR value of 2.5 had a sensitivity of >70% and specificity of >60% for MS. This cut-off identified larger number of adolescents with MS in different BMI categories (19.7% in normal weight, 51.7% in overweight, and 77.0% in obese subjects) as compared to the use of IDF or ATP III criteria for diagnosing MS. Odds ratio for having IR (HOMA-IR of >2.5) was highest with WHtR (4.9, p <0.0001) and WC (4.8, p <0.0001), compared to WHR (3.3, p <0.0001).

**Conclusions:** In Indian adolescents, HOMA-IR increased with sexual maturity and with progression from normal to obese. A HOMA-IR cut-off of 2.5 provided the maximum sensitivity and specificity in diagnosing MS in both genders as per ATP III and IDF criteria.

**Conflict of interest:**None declared.

## INTRODUCTION

Insulin resistance (IR) and the metabolic abnormalities related to IR have been associated with metabolic syndrome (MS), type 2 diabetes mellitus (T2DM) and cardiovascular disease (CVD) in adults and in the elderly. MS is now increasingly being recognized in children and adolescents (1,2). Childhood obesity which is increasing worldwide is well known for its association with IR (3).

IR is typically defined as decreased sensitivity or responsiveness to the metabolic actions of insulin, such as insulin-mediated glucose disposal and inhibition of hepatic glucose production. There are various tools used for quantifying insulin sensitivity and resistance directly (hyperinsulinemic euglycemic glucose clamping and insulin suppression tests) and indirectly [frequently sampled intravenous glucose tolerance test, oral glucose tolerance test, meal tolerance test, and homeostasis model of assessment-IR (HOMA)] (4). The utility of HOMA-IR in assessment of IR has been validated in children and adolescents (5,6). A HOMA-IR value of 2.5 is taken as an indicator of IR in adults (7), but the corresponding value in children and adolescents has not been established. Studies evaluating HOMA-IR in obese children and adolescents are few (5,8,9,10,11,12,13,14). Of these, a limited number are population-based studies, and the remaining are studies conducted on small samples. It is well known that the frequency of IR varies in the two sexes and among different ethnic groups. Since there are no studies from India identifying the cut-off levels for HOMA-IR in the adolescent population, we undertook this study to evaluate IR by HOMA-IR in Indian adolescents according to sexual maturation rating (SMR) and body mass index (BMI) categories and to establish cut-off values of HOMA-IR as an indicator of IR.

## METHODS

The subjects of a previously conducted survey in schools located in four different geographical zones of Delhi constituted the source from which the sample was selected (15). Nine hundred adolescents (300 each in obese, overweight and normal weight categories) in the age group 10-17 years were selected by computerized random number generation and were invited to take part in the study. A total of 691 apparently healthy adolescents consented to participate, and the group included 199 obese, 205 overweight, and 295 normal weight adolescents, as defined by the International Obesity Task Force criteria (16). These subjects underwent a detailed clinical, biochemical and hormonal evaluation and were found to be free of any systemic illness.

The study was conducted according to the guidelines laid down in the Declaration of Helsinki, and all procedures involving human subjects/patients were approved by the Institutional Human Ethics Committee of Institute of Nuclear Medicine and Allied Sciences, Timarpur, Delhi. Written informed consent was obtained from all subjects/patients. A prior consent for the study was taken from the school administration and from the parents. At the time of initiating the study, parents of each participant provided written informed consent for their ward’s participation. Assent from children was also obtained before drawing blood samples.

Height was measured to the nearest 0.1 cm using a stadiometer (a portable wall-mounted stadiometer (200 cm/78 inches) model DS045 manufactured by Narang Medical Ltd. Delhi) with the subject in the erect position, with his/her head held in Frankfurt horizontal plane. Weight was measured to the nearest 0.1 kg, without shoes and wearing light clothes, using an electronic digital weighing machine (EQINOX - Model EB6171, Equinox Overseas Private Limited, New Delhi). Height and weight measurements were taken twice, and the mean of two measurements was used to calculate BMI. Waist circumference (WC) was measured midway between the superior border of the iliac crest and the lowermost margin of the ribs at the end of normal expiration. Hip circumference was measured around the point with the maximum circumference over the buttocks, with feet fairly close together (about 12-15 cm apart) and weight equally distributed on each leg. Blood pressure was measured according to method described by the Seventh Report of the Joint National Committee (17).

The adolescents were given written instructions to fast for 12 hours, and compliance was assessed by interviewing the subjects and their parent(s) on the morning of the test. For the oral glucose tolerance test, the glucose load was calculated as 1.75 g/kg to a maximum of 75 g. Air tight packets of calculated glucose load were prepared for each child on the day prior to the test. After a 12-hour overnight fast, venous blood samples were drawn, and the participants were given the glucose load. Two-hour post-load samples were taken for determination of plasma glucose and serum insulin. During the interim period, the children were kept in the fasting state in the examination hall and did not indulge in any strenuous physical activity. Fasting and post-load plasma glucose levels were estimated on the same day and the remaining aliquots were stored at -20˚C until assayed.

The glucose oxidase–peroxidase method was used for measurements of plasma glucose (Clonital, Italy). Fasting serum total cholesterol, high-density lipoprotein (HDL) cholesterol, and triglycerides (TG) were estimated using an automated analyzer (Hitachi-902 fully automated biochemistry analyzer; Roche, Manheim, Germany) and commercial kits (Roche, Manheim, Germany). Serum insulin level was measured using commercial kits and an electrochemiluminiscence device (Elicsys, Roche Diagnostics), with a measurement range of 3.5-2083.5 pmol/L and a given normal value of 14.6-152.8 pmol/L. Intra-assay and inter-assay coefficients of variation were 4.3% and 3.4%, respectively. IR was calculated by using the HOMA model [HOMA-IR = fasting insulin (µIU/mL)*fasting glucose (mmol/L)/22.5] (4).

BMI was defined as the ratio of body weight to body height squared, expressed in kg/m2. WC cut-offs as proposed by Kuriyan et al (17) were used to identify children with a WC >90th centile (18). Waist/hip ratio (WHR) and waist/height ratio (WHtR) were calculated, and a WHR ratio of 0.9 in boys and 0.8 in girls (19) was taken as a cut-off for WHR and a WHtR of 0.5 (20) as a cut-off for WHtR. Using these cut off values, the subjects were divided into two groups. Hypertension was defined as a systolic (SBP)/diastolic blood pressure (DBP) greater than 90th centile for age and sex (21). SMR was determined by Tanner & White method (22,23)

MS in adolescents was defined by the International Diabetes Federation (IDF) criteria (24) [WC >90th percentile with any two of the parameters (TG ≥1.69 mmol/L, HDL <1.03 mmol/L, FPG >5.6 mmol/L, and BP >130/85 mmHg)] and Adult Treatment Panel III (ATP III) criteria (25) [abnormality in any of three parameters, namely WC > 90th percentile¸ dysglycemia¸ hypertension (>95th percentile for SBP or DBP), hypertriglyceridemia (>95th percentile), and low HDL (<5th percentile)]. Any degree of dysglycemia was defined by impaired fasting glucose (IFG) - fasting plasma glucose >100-125 mg/dL (>5.6-6.9 mmol/L), impaired glucose tolerance (IGT) - 2-hour post 75 glucose load plasma glucose 141-199 mg/dL (7.8-11.0 mmol/L) and DM - fasting plasma glucose ≥126 mg/dL (≥7.0 mmol/L) or post glucose plasma glucose ≥200 mg/dL (≥11.1 mmol/L) as per the definition provided by the American Diabetic Association (26). Recently, our group has published reference range for lipid profile in Indian adolescents in the age group 10-18 years, where the 95th percentile for TG in adolescents is above the adult limit of 150 mg/dL and the 5th percentile value for HDL is lower than 40 mg/dL (<1.03 mmol/L) (27). Hence, we have taken adult cut-off points in MS-ATP definition, which are that ≥1.69 mmol/L for TG, and <1.03 mmol/L for HDL.

The SPSS version 20.0 (SPSS Inc. Chicago, USA) was used for the statistical analyses. The data are presented as mean ± standard deviation or number (%), unless specified otherwise. All parametric data were analyzed by the independent student’s t-test in categorical groups. All non-parametric data were analyzed by chi-square test. Pearson’s correlation coefficient was calculated to assess the strength of relationship between lipid HOMA-IR and other parametric variables. Receiver operator characteristic (ROC) curves were plotted using HOMA-IR and presence or absence of metabolic abnormalities. Youden’s index was calculated by sensitivity - (1-specificity), obtained from co-ordinates of the curve. Highest value for Youden’s index was used to identify the cut-off value of HOMA-IR. A p-value of <0.05 was considered statistically significant.

## RESULTS

The basic characteristics of the study population are depicted in Table 1. The mean HOMA-IR of the study population was 2.83±2.05 (Boys: 2.77±1.98, Girls: 2.93±2.11). HOMA-IR showed no consistent pattern in relation to age and sex, showed the lowest value at age 13 years, then increased significantly to reach a peak at 17 years (Table 2).

**Figure 1:**
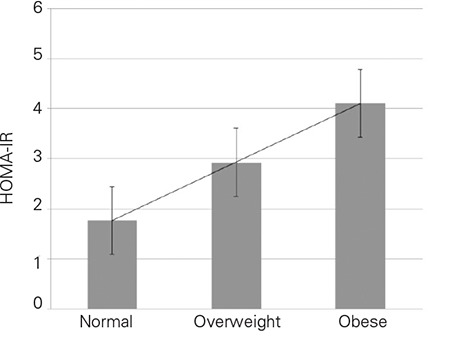
Homeostasis model of assessment insulin resistance (HOMA-IR) according to body mass index (BMI) categories

The HOMA-IR values increased progressively from normal weight to obese (Figure 1) in both sexes. Boys: normal weight 1.70±1.44 (95% CI 1.46-1.94) vs. overweight 2.67±1.41 (95% CI 2.40-2.94) vs. obese 4.39±2.14 (95%CI 3.95-4.83), p-value <0.0001 between all groups); Girls: normal weight 1.21±1.10 (95% CI 1.73-2.12) vs. overweight 3.19±2.02 (95% CI 2.79-3.60) vs. obese 4.19±2.52 (95% CI 3.69-4.69), p-value <0.0001 between all groups). The mean values of HOMA-IR were comparable among adolescent boys and girls as a group (p=0.78) and when divided according to BMI categories (Normal weight p=0.966; Overweight p=0.061; Obese p=0.168).

The mean HOMA-IR values progressively increased with increasing sexual maturation. There were 96 (13.8%) pre-pubertal (mean age 11.5±1.0, range 10-14 years) and 248 post- pubertal boys (mean age 13.7±1.8, range 10-17 years). HOMA-IR values were significantly higher in post-pubertal boys (2.92±2.08) compared to pre-pubertal boys (2.26±1.72, p=0.003). There was no significant difference in HOMA-IR values between pubertal stages 1-3. A significant difference in mean HOMA-IR values was observed between Tanner stages 1-2 and stages 4-5, in both boys and girls. The peak HOMA-IR values were reached at Tanner stage 5 in both sexes (Table 3).

The prevalence of MS in normal BMI, overweight, and obese adolescents was 1%, 18.4%, and 49% (p<0.00001), respectively, using the modified ATP III criteria and 0.3%, 13.6%, and 46.4% (p<0.00001), respectively, using the IDF definition. The HOMA-IR cut-off value for MS was determined by ROC curve. Although the HOMA-IR value of 2.0 showed maximum sensitivity for diagnosing MS as defined by IDF (86.3%) and ATP (85.1%), it had low specificity (46.4% and 47.7%, respectively). A sensitivity of >70% and specificity of >60% was indicated by HOMA-IR value of 2.5 (Table 4). According to this cut-off, the number of adolescents with IR in different BMI categories would be 58 (19.7%) in normal weight, 106 (51.7%) in overweight, and 147 (77.0%) in obese groups, respectively. When both techniques (definition of MS by IDF or HOMA-IR cut-off of 2.5) were combined, the yields for MS were higher: 59 (20.0%), 121 (58.7%), and 172 (90.0%) in normal, overweight, and obese group, respectively. Adolescents with any component of MS had significantly higher HOMA-IR values than those without (Table 4).

HOMA-IR was positively correlated with the majority of anthropometric and biochemical parameters (Table 5). The odds ratio for having IR (HOMA-IR of >2.5) was greater with high WHtR [4.9 (95% CI 3.6-6.8, p<0.0001)] and WC [4.8 (95% CI 3.5-6.7, p<0.0001)] compared to WHR [3.3 (95% CI 2.4-4.8, p<0.0001)]. 

## DISCUSSION

The increase in incidence and prevalence of T2DM and its cardiovascular complications is probably a consequence of the global epidemic of obesity. The rising prevalence of obesity in the Indian child and adolescent population is a cause of concern (28,29,30) as it not only predisposes to adult obesity but also increases the likelihood of negative health consequences (31). Commensurate with the rising trend of adolescent obesity is the rising prevalence of IR and MS in this population (32,33,34,35). Several studies from different populations were conducted to establish HOMA-IR cut-off values for MS in adolescents (5,6,13,14). At present, however, there are no data defining the cut-off values of HOMA-IR for Indian adolescents. A study from the southern part of India demonstrated a correlation between HOMA-IR and glucose intolerance (36). This present study is the first large scale work which analyzed HOMA-IR values in obese, overweight, and normal weight adolescents in both sexes, also taking into account age, pubertal status, and metabolic abnormalities associated with MS.

In our study, the mean HOMA-IR values showed inconsistent pattern according to age with no significant difference among girls in different age groups in contrast to boys, who showed lowest levels at 13 years and peak HOMA-IR values at 17 years of age. A study among healthy children from Spain showed a progressive rise in HOMA-IR with age for both boys and girls (37). The Early Bird Diabetes study from UK had prospectively evaluated HOMA-IR in healthy children aged 5-14 years (9) and found IR to start rising from mid-childhood (~ 7 years), a few years before puberty. The present study comprising adolescents between 10-17 years, being cross-sectional in design, may have missed an early rise of IR, which may have stabilized after pre-pubertal years. Also, lack of a correlation of HOMA-IR with age may possibly be related to the heterogeneity of pubertal status in the same chronological age groups, especially among girls.

In this study, as expected, both boys and girls showed a progressive increase in mean HOMA-IR values with increasing BMI. A significant difference among the HOMA-IR values of normal weight, overweight, and obese adolescents was observed. High values for HOMA-IR in obese subjects compared to normal adolescents [Mean HOMA-IR: 4.93 (95% CI 4.56-5.35) vs. 2.30 (2.21-2.39), respectively], has also been validated in a large study among adolescents from USA (8).

There is no consensus on HOMA-IR cut-off values for identifying MS in adolescents. In the current study, a HOMA-IR cut-off of 2.5 provided adequate sensitivity and specificity in diagnosing MS in both boys and girls as per ATP III and IDF criteria. There are no previous studies from India which have tried to establish HOMA-IR cut-offs to identify MS in this subset of population. HOMA-IR values ranging from 2.22 to 3.16 have been reported as cut-off for identifying IR in various studies (5,12,13,38), but all these studies were conducted on small samples of obese children and adolescents and hence, are likely to report higher cut-off values. The cut-off values derived in our study are more likely to be applicable as they have been derived from a large cohort with a homogenous mix of normal-weight, overweight, and obese adolescents.

IR has been implicated in causation of MS; this may imply that metabolic abnormalities associated with MS are the end result of long-term IR (38). This is further reiterated by the results of the present study, where HOMA-IR cut-off identified a large number of normal-weight and overweight adolescents with IR who otherwise would have been missed by the IDF and ATP III criteria. A similar observation was made by a study from Turkey (13). These children may be at risk of future development of MS. This assumes significance for children and adolescents from the Indian subcontinent, since these populations were found to be more insulin resistant than their Caucasian counterparts (39). This is further substantiated by our study, where 77% of obese adolescents had IR compared to about 52% in a study among US adolescents (8). Hence, intervention in the group of adolescents with underlying IR without metabolic abnormalities may prevent development of MS. However, this strategy requires validation in future studies.

In this study, HOMA-IR showed significant correlations with BMI, WC and WHtR, while the correlation between WHR and HOMA-IR was poor. Other studies including one from India (35) also observed the strongest correlation of HOMA-IR with WC (35,40,41,42). Another parameter which has shown good correlation with HOMA-IR in our study is WHtR. A similar result was reported in a large study from Europe (43). All these studies have shown that WC and WHtR are good predictors of IR in adolescents and can be used to identify at risk individuals. Amongst the various biochemical parameters, elevated TG was correlated with HOMA-IR, whereas HDL did not show a significant correlation. HOMA-IR was significantly higher in those adolescents who were positive for components of MS.

The strength of our study is the large number of adolescents in the sample with comparable distribution in all BMI categories. Its limitation is absence of longitudinal follow-up.

In conclusion, this study has shown that HOMA-IR is a valuable tool in identifying adolescents with MS. A HOMA-IR cut-off of 2.5 provided the maximum sensitivity and specificity in diagnosing MS in both genders as per ATP III and IDF criteria. This cut-off value may also serve to identify adolescents at high risk of future MS and who are amenable to early intervention. Among anthropometric parameters, WC and WHtR are the two anthropometric parameters that correlated best with IR.

## ACKNOWLEDGEMENT

We are thankful to Dr Namita Mahalle, Department of Pathology, Deenanath Mangeshkar Hospital and Research Centre, Erandawane, Pune for providing help in statistical analysis. 

## Figures and Tables

**Table 1:**
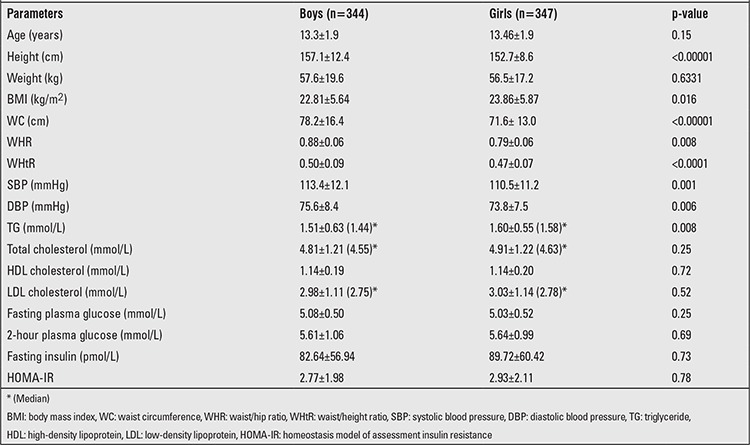
Basic characteristics of the study population

**Table 2:**
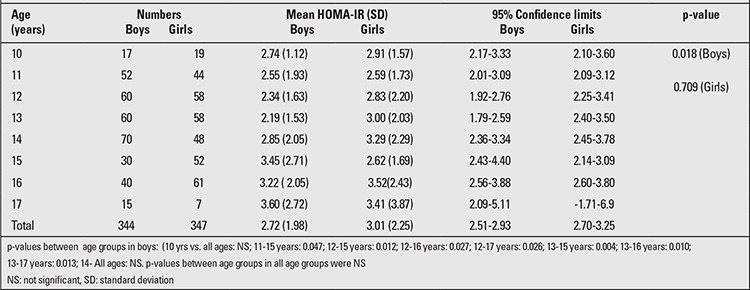
Homeostasis model of assessment insulin resistance (HOMA-IR) according to age in both sexes

**Table 3:**
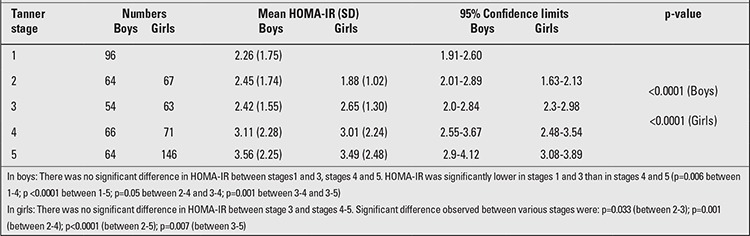
Homeostasis model of assessment insulin resistance (HOMA-IR) mean values by stages of pubertal development

**Table 4:**
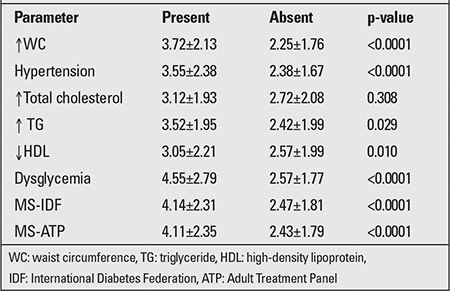
Homeostasis model of assessment insulin resistance(HOMA-IR) by various metabolic syndrome (MS) parameters

**Table 5:**
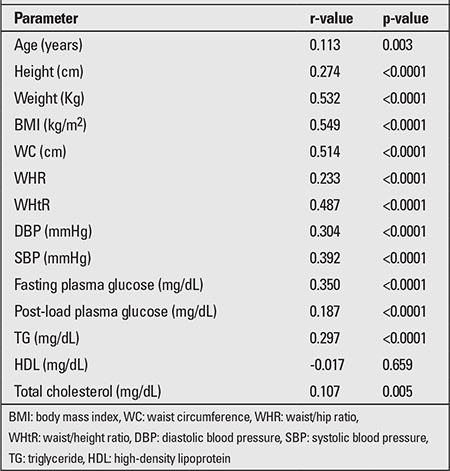
Correlation of various parameters with homeostasis model of assessment insulin resistance (HOMA-IR)
